# Preliminary study on the effects of enrofloxacin, flunixin meglumine and pegbovigrastim on *Mycoplasma bovis* pneumonia

**DOI:** 10.1186/s12917-019-2122-3

**Published:** 2019-10-26

**Authors:** Katarzyna Dudek, Dariusz Bednarek, Roger D. Ayling, Anna Kycko, Michał Reichert

**Affiliations:** 1 Department of Cattle and Sheep Diseases, National Veterinary Research Institute, 57 Partyzantów Avenue, 24-100 Pulawy, Poland; 2 Mycoplasma Team, Animal and Plant Health Agency (Weybridge), Woodham Lane, Addlestone, Surrey, KT15 3NB UK; 3 Department of Pathology, National Veterinary Research Institute, 57 Partyzantów Avenue, 24-100 Pulawy, Poland

**Keywords:** *Mycoplasma bovis*, Cattle, Fluoroquinolones, Flunixin, Pegbovigrastim

## Abstract

**Background:**

*Mycoplasma bovis* is a causative agent of disease in cattle causing many clinical conditions. Currently there are no commercial *M. bovis* vaccines in Europe and treatment is difficult with decreased antimicrobial susceptibility of *M. bovis* field isolates. Using an *M. bovis* calf infection model the effectiveness of enrofloxacin given alone; in combination with flunixin meglumine, a nonsteroidal anti-inflammatory drug; and a group with an additional treatment of pegbovigrastim, an immunostimulator, was evaluated.

**Results:**

Enrofloxacin given alone stimulated a strong immune response, reduced the clinical manifestation and lung lessions of the *M. bovis* infection. In contrast the combination therapy appeared ineffective.

**Conclusions:**

In this experiment enrofloxacin given alone appeared to be the most effective treatment of the *M. bovis* affected calves, whereas co-administration with flunixin meglumine, and pegbovigrastim was not beneficial in this trial.

## Background

*Mycoplasma bovis* causes disease and many clinical signs in cattle which includes bronchopneumonia, arthritis and mastitis. It is also known as a causative agent of bovine respiratory disease (BRD) and is responsible for huge economic losses [1, 2]. Currently there are no commercial vaccines against *M. bovis* infections. In some countries autogenous vaccines are used; and work continues to develop a suitable commercial vaccine as a principal strategy for control of *M. bovis* infections [3–5]. The β-lactam antimicrobials (penicillins, cephalosporins) mode of action is against the cell wall, therefore these antimicrobials are ineffective against the cell wall-less *M. bovis*. In addition many in vitro studies on European field *M. bovis* isolates show increasing trends in antimicrobial resistance, especially for the tetracyclines and macrolides [6–8], with some isolates appearing resistant to most classes of antimicrobials that have been licenced for use against *M. bovis* in cattle [9]. In a recent European collaborative study in vitro antimicrobial sensitivities, minimum inhibitory concentrations (MICs), were obtained for 156 *M. bovis* isolates against four classes of antimicrobials including the fluoroquinolones, macrolides, amphenicols and tetracyclines. The study showed the lowest MIC_50_/MIC_90_ values for fluoroquinolones, whereas high values indicating antimicrobial resistance was observed for some macrolides including the newer generation macrolides tulathromycin and gamithromycin [5]. Therefore the fluoroquinolones may be the most effective antimicrobials to treat *M. bovis* infections [5, 8] however they are a class of antimicrobials that should be used as a last resort. The use of antimicrobials in animals is increasingly controversial, as a reduction in their use is recommended to reduce the formation of resistance and possible adverse impact on antimicrobial control of human diseases. The use of nonsteroidal anti-inflammatory drugs (NSAIDs) in combination with antimicrobials may enhance their effectiveness and reduce the amount of antimicrobial required and subsequently avoid the development of resistance. The antipyretic effect of NSAIDs such as flunixin meglumine, carprofen, ketoprofen or meloxicam are often used in combination with antibiotherapy to treat various cattle diseases [10–12]. To improve innate immunity some immunostimulators have been used to support traditional antimicrobial therapy in cattle. One such non-specific activator of cattle immunity is pegbovigrastim, a modified form of cytokine bound to polyethylene glycol that stimulates bovine granulocyte colonies, which was used successfully in periparturient dairy cows [13].

This study evaluates the efficacy of three therapy models in the treatment of calves infected with an *M. bovis* field strain. Treatment included: a) enrofloxacin, a fluoroquinolone antimicrobial; b) enrofloxacin combined with flunixin meglumine, a NSAID; c) enrofloxacin, with flunixin meglumine and pegbovigrastim, an immunostimulator. The aim was to determine an effective approach to controlling *M. bovis* infections in cattle.

## Results

### *Pasteurellaceae* species and antibodies to BHV1, BVDV, BRSV and PI3V

Before the experiment no *Pasteurellaceae* species were isolated from deep nasal swabs. Analysis of blood samples showed that two calves were positive for BHV1 antibodies, seven for BVDV, twenty for BRSV and twenty-one for PI3V antibodies, respectively. No seroconversion for specific antibodies to these viruses was observed during the experiment indicating a lack of active viral infections.

### Minimal inhibitory concentrations

The lowest MIC values (0.25 μg/ml) were obtained for enrofloxacin therefore this antimicrobial was used for the calf treatment.

### Clinical observations

The calves dosed with *M. bovis* showed increasing clinical signs consistent with an *M. bovis* infection. Before treatment early respiratory signs of infection with nasal discharge and some coughing were present in all of the dosed calves, with no clinical signs in the NC group (Additional file 1: Table S1). On day 11 post the first infecting dose one PC calf was sacrificed due to a severe *M. bovis* infection (Additional file 2: Table S2).

The day after the various treatments, experiment day 10, the clinical status of the E1, E2 and E3 calves was visibly improved, with a reduction in nasal discharge and coughing when compared to the PC group, however the improvement was most apparent in the E1 group.

### *M. bovis* antigen

#### Nasal swabs

*M. bovis* was not detected in any calf nasal swabs before the experiment. On day 3 post the first infecting dose it was detected in all of the experimental calves and intermittently from all of the experimental calves on subsequent sampling. Post treatment *M. bovis* antigen was detected in the lowest rates in the E3 group compared to the PC group. The NC group was negative for *M. bovis* antigen.

### Post mortem

All six calves from each experimental and the PC groups and two calves from the NC group were examined post mortem. *M. bovis* antigen was detected from the lungs of all the experimental and PC calves, however in one calf from the E1 group the *M. bovis* antigen Val was very low (negative; 1.99%). In this group the *M. bovis* antigen was also detected with the lowest mean Val (28.12%) from the trachea. The lungs of the NC calves according to the test interpreting were unequivocally free for *M. bovis* antigen, however a very low (negative) insignificant Val (5.1%) was detected in one calf from the MLN and in the same animal an insignificant Val (22.2%) was also recorded within the trachea. The mean Val of *M. bovis* antigen in all of the examined organs of the E1 group was lower than the PC group, whereas for the E2 and E3 groups it was reduced only in the trachea or MLN.

### Immunohistochemistry

**Fig. 1:**
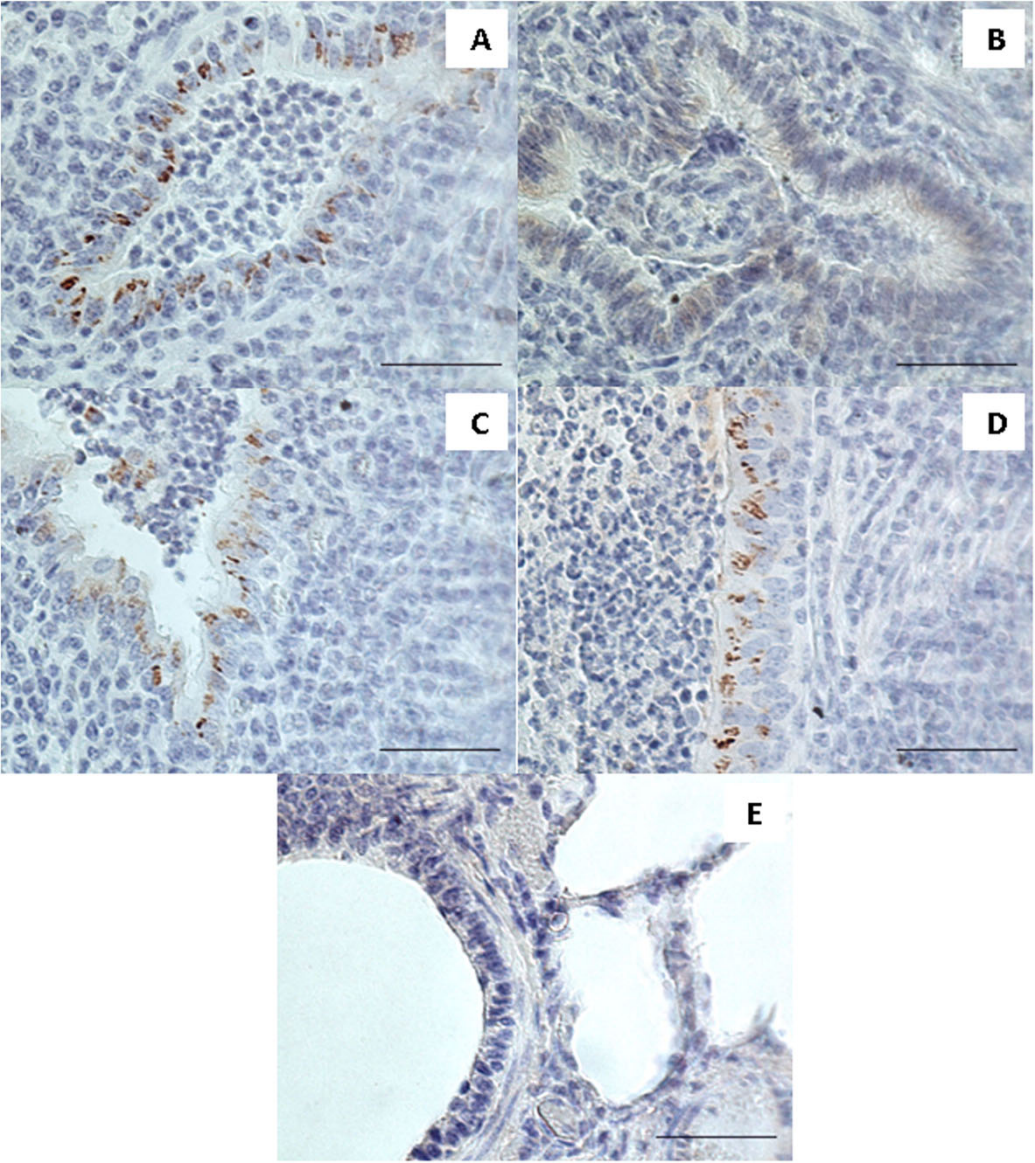
Immunohistochemistry. Positive labelling for *M. bovis* visible as dark brown granular aggregates was observed in epithelial cells of bronchioli in the lung of the positive control calf infected with *M. bovis* (**a**), the lung of the calf infected with *M. bovis* and received enrofloxacin combined with flunixin meglumine (**c**) and the lung of the calf infected with *M. bovis* that received enrofloxacin combined with flunixin meglumine and pegbovigrastim (**d**). The lung of the calf infected with *M. bovis* and received enrofloxacin alone showing lack of specific granular immunolabelling with faint diffuse light brown staining visible in bronchiolar epithelium (**b**). The lung of a negative control calf showing lack of labelling for *M. bovis* (**e**). Bar = 50 μm

Immunohistochemical analysis of the PC group showed *M. bovis* in the bronchiolar epithelial cells in the lung areas with histopathological changes characteristic for bronchiolitis. The positive reaction manifestated itself as dark-brown granules aggregating in the cytoplasm of the epithelial cells (Fig. 1a). In the lung sections of E1 group, there were singular bronchioli displaying the immunolabelling, while in large parts of the tissues there was no staining or only faint diffuse reaction observed in the epithelium (Fig. 1b) and occasionally in an inflammatory exudate in the bronchial lumena. In one lung from the E1 group, there was positive immunostaining visible in bronchioli in the whole section. In the E2 group, in three cases, there was strong labelling of *M. bovis* visible in bronchioli, similar to the one in the PC group (Fig. 1c). In the other three cases, the staining was observed in few bronchioli. In the E3 group with just one exception all of the lung sections had strong positive reactions visible in the bronchiolar epithelium in the inflammatory areas (Fig. 1d), the one exception had no characteristic staining. In all of the sections from the *M. bovis*-infected animals there was no positive reaction for the antigen observed within the necrotic areas of the tissues. The lung sections from the NC group had no immunolabelling for *M. bovis* (Fig. 1e).

### Anti-*M. bovis* antibodies

**Fig. 2:**
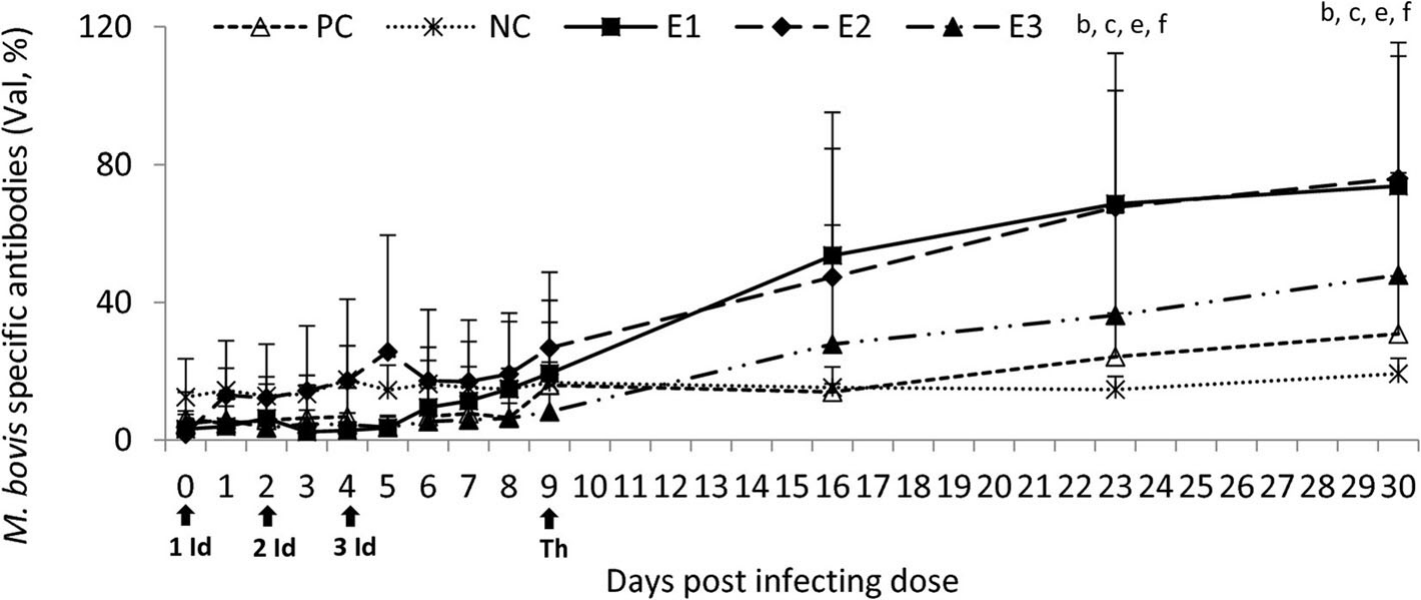
Anti-*M. bovis* specific antibodies in the sera of the experimental and control calves post the treatment. NC, negative control; PC, positive control; E1, group received enrofloxacin alone; E2, group received enrofloxacin combined with flunixin meglumine; E3, group received enrofloxacin combined with flunixin meglumine and pegbovigrastim; 1, 2, 3 Id – the 1st, 2nd and 3rd infecting dose of *M. bovis*; Th – day of therapy starting; b, *P* < 0.05 between groups E1 and NC; c, P < 0.05 between groups E2 and NC; e, P < 0.05 between groups E1 and PC; f, P < 0.05 between groups E2 and PC

Post treatment a progressive increase in the *M. bovis* specific antibody percentage in each experimental group was observed compared to the NC and PC groups, visibly marked in the E1 group. On days 23 and 30 it was increased also in the PC group compared to the NC group in which *M. bovis* specific antibodies were stable throughout the study. Statistically significant differences between the E1 and NC/PC as well as between E2 and NC/PC groups were observed on days 23 and 30 (Fig. 2).

### Immunological analyses

**Fig. 3:**
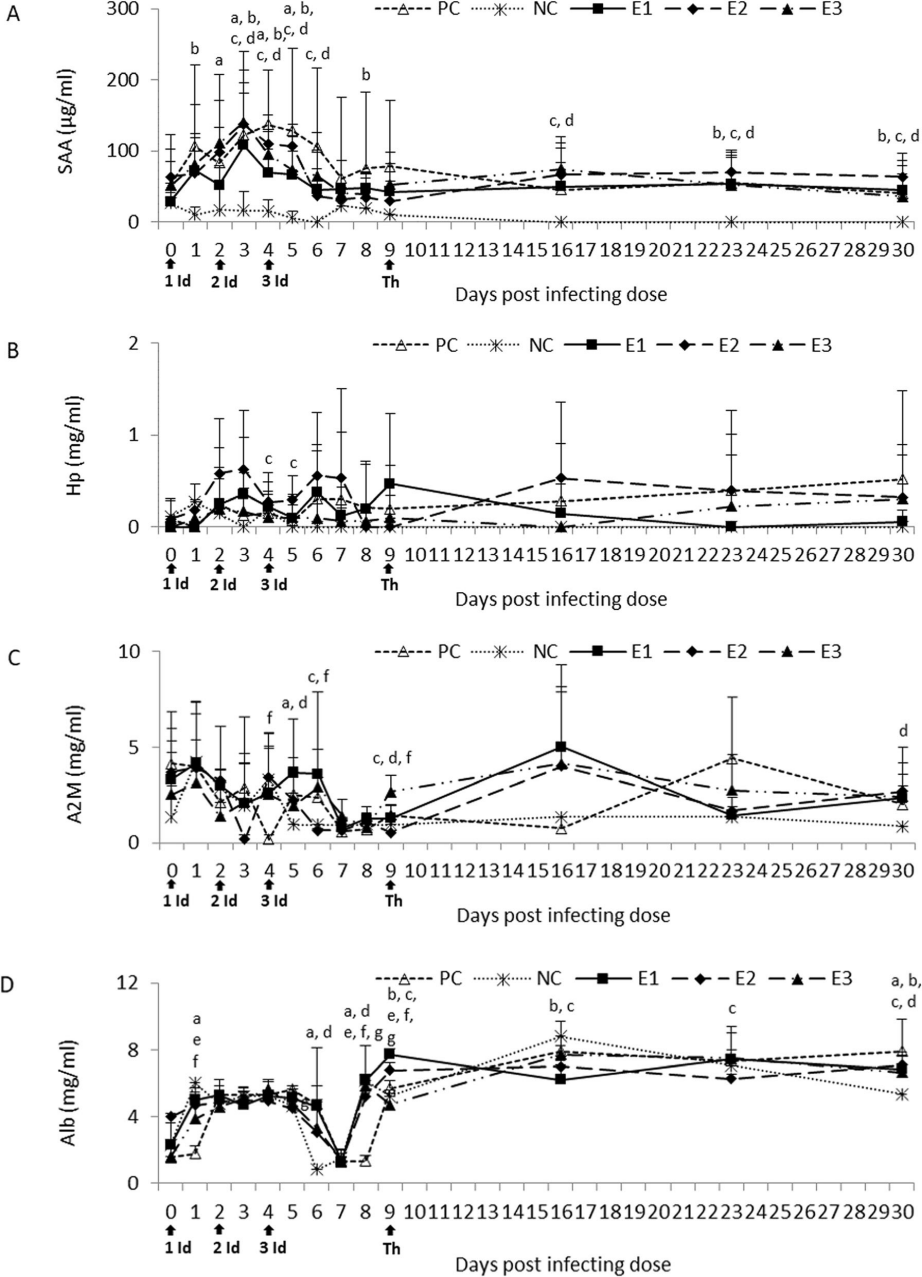
SAA (**a**), Hp (**b**), A2M (**c**) and Alb (**d**) concentration in the sera of the experimental and control calves post treatment. NC, negative control; PC, positive control; E1, group received enrofloxacin alone; E2, group received enrofloxacin combined with flunixin meglumine; E3, group received enrofloxacin combined with flunixin meglumine and pegbovigrastim; 1, 2, 3 Id – the 1st, 2nd and 3rd infecting dose of *M. bovis*; Th – day of therapy starting; a, P < 0.05 between groups PC and NC b, P < 0.05 between groups E1 and NC; c, P < 0.05 between groups E2 and NC; d, P < 0.05 between groups E3 and NC e, P < 0.05 between groups E1 and PC; f, P < 0.05 between groups E2 and PC; g, P < 0.05 between groups E3 and PC

Post infection a distinct increase in the SAA concentration in all of the infected groups was observed compared to the NC group with statistically significant values on days 1–6 and 8. One week after therapy started it was higher in all of the experimental groups compared to the NC and PC groups with statistically significant differences between E2/E3 and NC. On weeks 2 and 3 post therapy the SAA concentration remained significantly higher in all of the experimental groups than the NC group and slightly higher or similar to those observed in the PC group (Fig. 3a).

Calves infected with *M. bovis* caused general stimulation in the Hp concentration in all experimental and PC groups compared to the NC group. Statistically significant differences between the E2 and NC groups were observed on days 4 and 5. Throughout the experiment the NC group had low Hp concentration while the Hp concentration in the PC group continued to increase. Following therapy the Hp of E1 (the fluoroquinolone treated calves) declined to the level of the NC group. The HP of the E2 group increased in the week following therapy and then declined below that of the PC group, whilst the E3 group declined in the first week following therapy and then increased to the same level as the E2 group (Fig. 3b).

At day 5 post the initial infection the A2M concentration in all of the experimental and PC groups was visibly higher than the NC group with statistically significant difference between the PC/E3 and NC group. However 1 week post therapy these groups had higher A2M than the PC and NC groups. The NC group A2M remained at a consistently low level after day 5, whilst the other groups fluctuated but were at a similar higher level than the NC group at the end of the experiment with statistically significant values for the E3 group (Fig. 3c).

In response to the first infecting dose all of the experimental and PC groups had a lower Alb (BSA) concentration compared to the NC group with statistically significant values for the PC group. However on day 2 post the third infecting dose it was visibly higher in these groups compared to the NC group with significant differences for the PC and E3 group. For the week post therapy the BSA for the PC and NC increased, as did the E3 group, but to a lesser extent, whilst the E1 and E2 groups declined. Statistically significant differences were observed at that time between the E1/E2 and NC group. At week 3 post therapy starting the BSA concentration in all experimental groups was significantly higher than the NC group but its values were lower than the PC group (Fig. 3d).

**Fig. 4:**
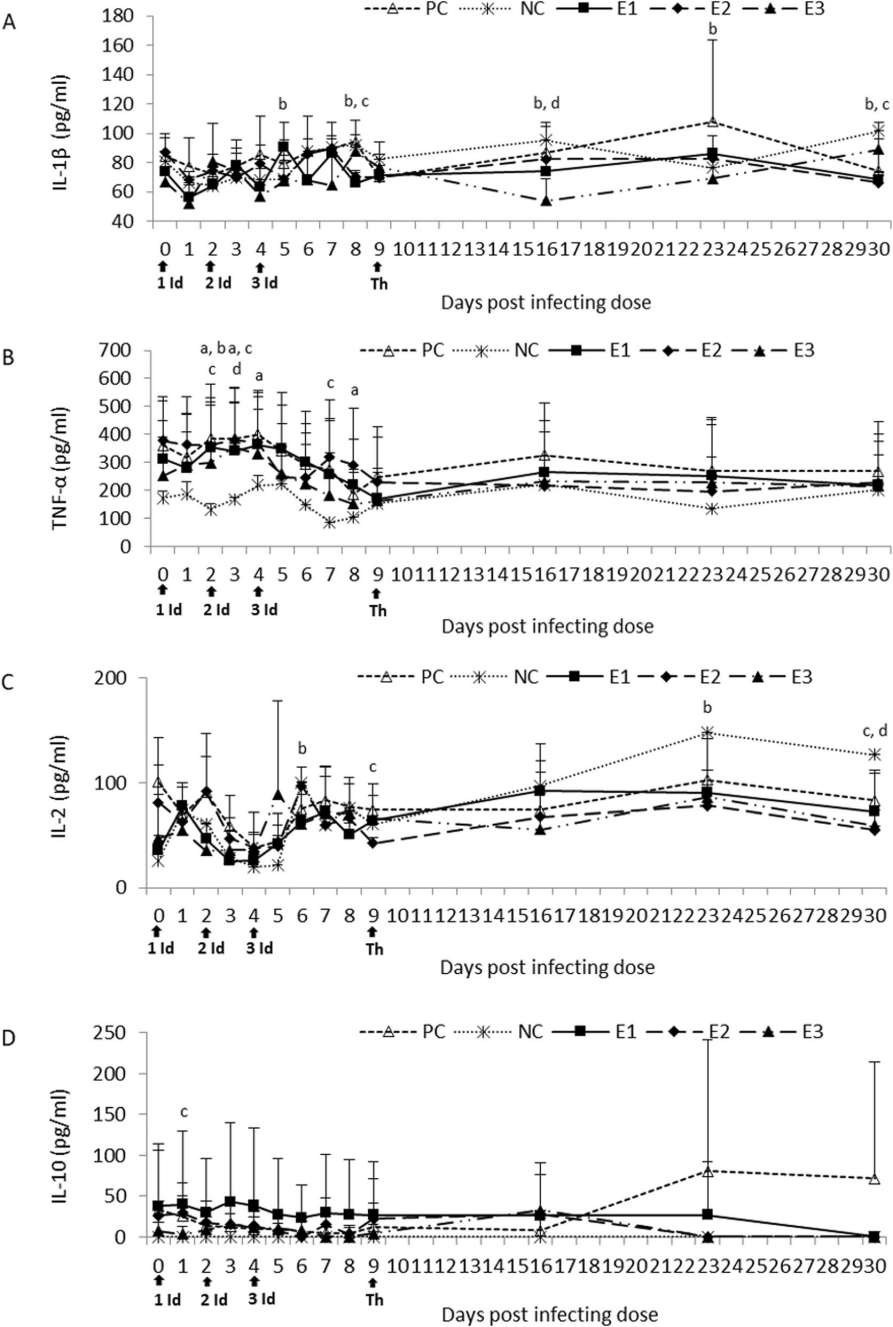
IL-1β (**a**), TNF-α (**b**), IL-2 (**c**) and IL-10 (**d**) concentration in the sera of the experimental and control calves post the treatment. NC, negative control; PC, positive control; E1, group received enrofloxacin alone; E2, group received enrofloxacin combined with flunixin meglumine; E3, group received enrofloxacin combined with flunixin meglumine and pegbovigrastim; 1, 2, 3 Id – the 1st, 2nd and 3rd infecting dose of *M. bovis*; Th – day of therapy starting; a, P < 0.05 between groups PC and NC b, P < 0.05 between groups E1 and NC; c, P < 0.05 between groups E2 and NC; d, P < 0.05 between groups E3 and NC

The IL-1β concentration was slightly increased in all the experimental and PC groups at different time points after challenge compared to the NC group with statistically significant values for the E1 and E2 group. However on week one post therapy starting the IL-1β concentration was lower in all of the experimental groups compared to the NC and PC groups with significant differences between the E1/E3 and NC group. On week 2 post therapy starting it was comparable to the NC group and had significantly lower values than the PC group, with the exception of the E1 group in which significantly higher values were observed. However on week 3 post therapy starting the IL-1β concentration in the E1 and E2 groups was significantly lower than the NC group (Fig. 4a).

Initially the TNF-α concentration was much lower in the NC group than the other groups, but all groups showed a decreasing concentration post infection (experiment day 5). Statistically significant differences were observed for all infected groups compared to the NC group at different time points post infection. Subsequently post therapy the TNF-α concentrations in the treated groups were lower than the PC group and similar or higher than the NC group (Fig. 4b).

Post infection a general decrease in the IL-2 concentration was observed in the E1 and PC groups compared to the NC group with statistically significant values for the E1 group on day 6. In the E2 and E3 groups the concentration was generally increased compared to the NC group however on the day therapy started significantly lower values were observed in the E2 group. On day one post the third infectious dose the IL-2 concentration was increased in all of the experimental and PC groups compared to the NC group. However on week one post therapy starting the concentration in the E1 group was comparable to the NC group, whereas in the other experimental groups it was lower or comparable to the PC group. On weeks 2 and 3 the IL-2 concentration in all of the experimental groups was lower than NC group and it was slightly less than the PC values. Statistically significant differences were observed between the E2/E3 and NC group on week 3 post therapy starting (Fig. 4c).

Infecting the calves did not cause significant changes in the IL-10 concentration when compared to the NC group, with the exception of day one for the E2 group. However on week one post therapy it was slightly higher in all of the experimental groups compared to the NC and PC group. This increase continued in the E1 group on week 2 post therapy starting. However by week 3 the IL-10 concentration was not detectable in the experimental and NC groups, whereas the PC group had increased values (Fig. 4d).

### Gross pathology

**Fig. 5:**
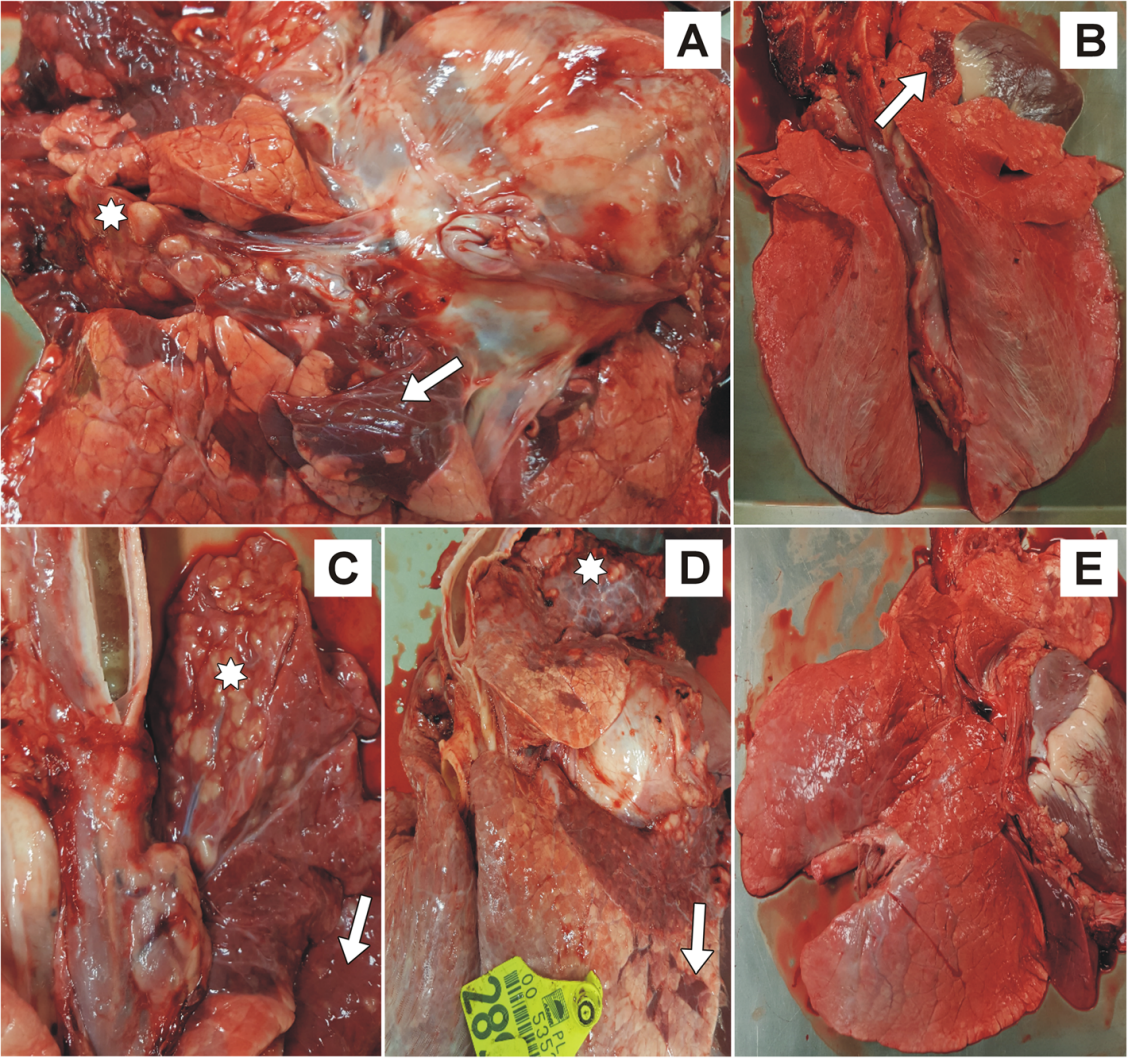
The lungs of treated and control calves. The lung of a positive control calf infected with *Mycoplasma bovis* (**a**): extensive caseous necrosis (star) and marble-like lesions (lobular consolidation, arrow). Lung from a calf infected with *M. bovis* and received enrofloxacin alone (**b**): a slight lobular consolidation (arrow). The lung of the calf infected with *M. bovis* and received enrofloxacin combined with flunixin meglumine (**c**): extensive caseous necrosis (star) and marble-like lesions (arrow). The lung of a calf infected with *M. bovis* that received enrofloxacin combined with flunixin meglumine and pegbovigrastim (**d**): extensive caseous necrosis (star) and marble-like lesions (arrow). The lung of a negative control calf (**e**): no lesions

Gross pathology of all the PC calves showed lung lesions typically associated with *M. bovis* infection. Extensive caseous necrosis and marble-like lesions (lobular consolidation) most often located in the apical and cardiac lobes was observed (Fig. 5a). Five PC calves had enlarged MLN’s and abundant mucous exudate was present in the trachea of four calves.

A slight lobular consolidation was observed in the lungs (Fig. 5b) of five E1 calves and one calf had caseous necrosis in the apical lobe. An enlarged MLN was observed in just one other calf. Two calves, one with the lung necrosis, had abundant mucous exudate in the trachea.

In four of the E2 calves extensive caseous necrosis and marble-like lesions in the lungs were seen (Fig. 5c). The lungs of two E2 calves showed slight lobular consolidation. Five E2 calves had enlarged MLN’s and an abundant mucous exudate in the trachea.

The gross lung pathology of five E3 calves showed extensive caseous necrosis and marble-like lesions (Fig. 5d), whereas in one calf just slight consolidation in the apical and cardiac lobes was observed. Three calves had enlarged MLN’s and five had abundant mucous exudate in the trachea.

No lesions were present in the NC calves (Fig. 5e).

### Histopathological analysis

**Fig. 6:**
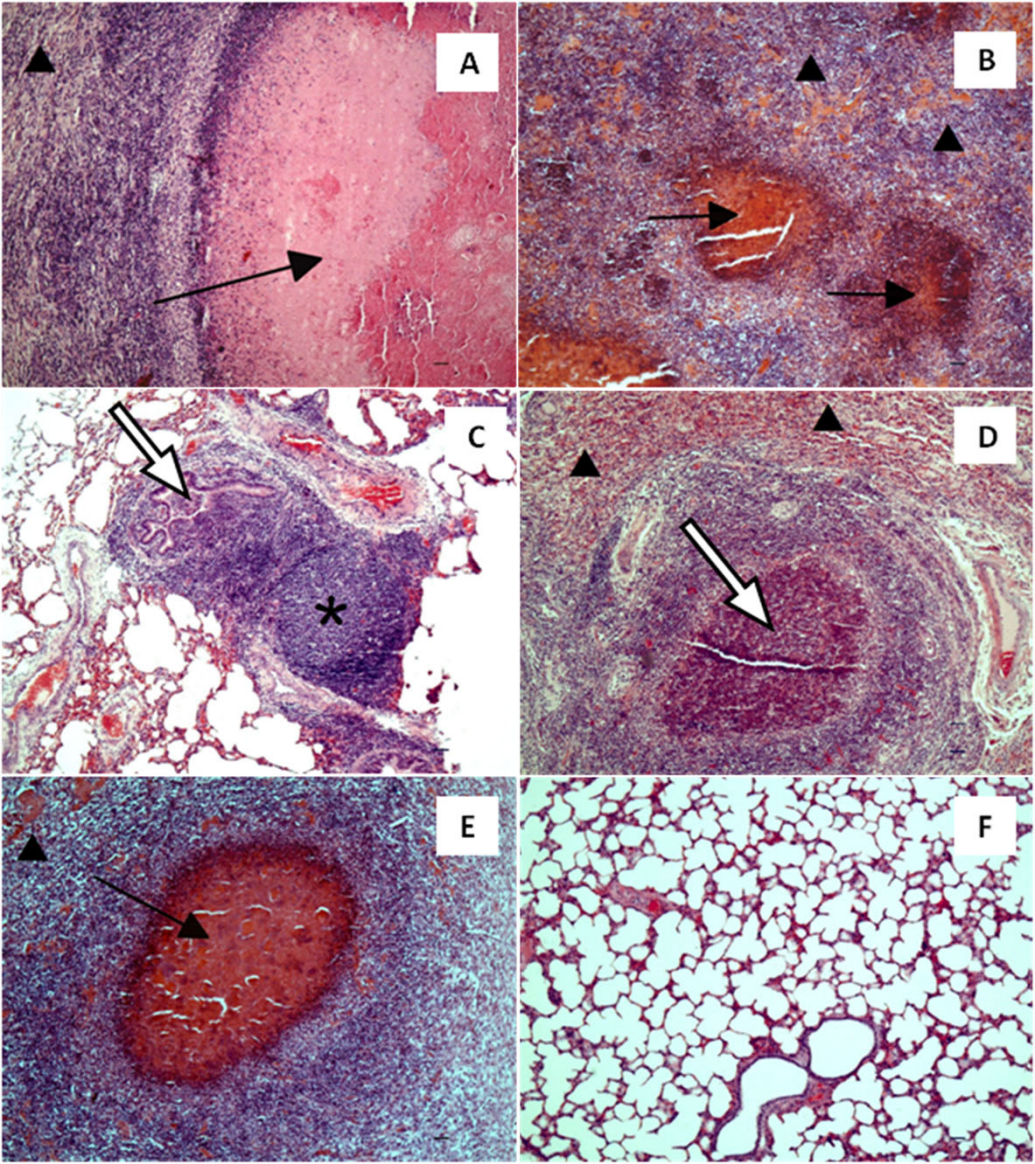
The lungs of treated and control calves. The lung of a positive control calf infected with *Mycoplasma bovis* (**a**): extensive caseous necrosis with amorphic eosinophilic core surrounded by macrophages and lymphocytes infiltrating surrounding lung parenchyma (black arrow); prominent atelectasia (arrow head). (**b**): multifocal inflammatory infiltrations with necrotic on centres containing macrophages and neutrophils, surrounded by lymphoidal cells (black arrow); prominent atelectasia (arrow head). The lung of the calf infected with *M. bovis* and received enrofloxacin alone (**c**): mild hyperplasia of bronchiolar-associated lymphoid tissue (star), as well as accumulation of neutrophils and macrophages in bronchiolar lumens (white arrow). The lung of a calf infected with *M. bovis* and received enrofloxacin combined with flunixin meglumine (**d**): extensive diffuse infiltration of neutrophils and macrophages and occasional foci of necrotic cells surrounded by macrophages and lymphoid cells (white arrow); prominent atelectasia (arrow head). The lung of the calf infected with *M. bovis* that received enrofloxacin combined with flunixin meglumine and pegbovigrastim (**e**): focal caseous necrosis (black arrow); prominent atelectasia (arrow head). The lung of a negative control calf (**f**). HE, Bar = 50 μm

Histopathological examination of the PC calves lungs revealed extensive areas of caseous necrosis with amorphic eosinophilic core surrounded by macrophages and lymphocytes infiltrating surrounding lung parenchyma (Fig. 6a). Bronchial lumens were filled with numerous neutrophils many of which were necrotic. The lesions were accompanied by prominent atelectasia (Fig. 6b). MLN’s showed massive hyperaemia and had necrotic cells in the follicles.

In the E1 calves moderate changes in lung sections were observed, characterized by mild hyperplasia of bronchiolar-associated lymphoid tissue (BALT), as well as an accumulation of neutrophils and macrophages in bronchiolar lumens (Fig. 6c). MLN’s were characterized by prominent hyperplasia of follicle centres with numerous macrophages containing necrotic material.

The lungs of the E2 calves were characterised by extensive diffuse infiltration of neutrophils and macrophages and occasional foci of necrosis surrounded by macrophages and lymphoid cells. There were necrotic neutrophils visible in the bronchioli as well as moderate BALT hyperplasia, accompanied by compression and atelectasis of alveoli and accumulation of eosinophilic edematous fluid in the airways (Fig. 6d). MLN’s showed mild hyperplasia of follicle centers.

Histopathological changes in lungs in the E3 calves involved prominent multifocal areas of caseous necrosis, resembling the lesions observed in the PC calves (Fig. 6e). Similarly to the E2 calves, there were diffuse infiltrations of neutrophils and macrophages in lung parenchyma accompanied by alveolar atelectasis and accumulation of oedematous fluid in alveoli. There was an exudate containing neutrophils visible in the bronchioli as well as mild BALT hyperplasia. Moderate hyperplasia of follicle centers containing necrotic cells and hyperaemia were observed in the MLN’s.

No changes were observed in the lung and MLN’s of the NC calves (Fig. 6f).

## Discussion

This is the first study that has evaluated the effectiveness of enrofloxacin administered alone and in combination with flunixin meglumine, and combined with pegbovigrastim in an *M. bovis* infection animal model.

Some beneficial effect of using combination therapy in the treatment of calves affected by BRD included the co-administration of florfenicol and flunixin meglumine in one injection has been reported [10, 11]. A standardized therapeutic protocol based on co-administration of two antimicrobials (tulathromycin and oxytetracycline hydrochloride) combined with carprofen was also proposed for the treatment of calves diagnosed with otitis media and media-interna, mostly caused by *M. bovis* [12]. A study using clinically healthy animals administered with enrofloxacin and flunixin meglumine in calves reported that the mean time-antimicrobial activity of enrofloxacin persisted until 6 h and its binding affinity to serum proteins was low [14]. The therapeutic effect of enrofloxacin in calves co-infected with *M. bovis* and *Mannheimia haemolytica* was previously shown [15], and in vitro studies have indicated enrofloxacin’s efficacy [5, 8]. Hence the selection of treatments applied in this study, combined with enrofloxacin which had the lowest MIC values of the 12 antimicrobials tested against the infecting *M. bovis* isolate was used.

In this study the most beneficial effect of the therapy was observed in the E1 group administered with enrofloxacin alone. Previously Abo El-Sooud and Al-Anati [14] reported that intramuscular administration of flunixin meglumine prior to enrofloxacin injection changed some of its pharmacokinetic parameters reducing the serum activity of enrofloxacin. However they showed that intravenous administration did not have the same effect. Although we used different administration routes for the two drugs the co-administration of flunixin may explain why the combined treatment model was ineffective in the E2 and E3 groups, however distribution properties may differ in diseased animals.

In the E1 group, post treatment, a strong specific humoral response was observed which coincided with a distinct reduction in clinical signs associated with mycoplasmal infection. In the E3 and PC groups where clinical changes were more expressed, only weak humoral responses were observed. Thus there are strong indications that during an *M. bovis* infection an improvement of the animal health status is correlated with a strong humoral response. Additionally, in the E1 group higher *M. bovis* nasal shedding was observed following treatment, which may be related to lower lung colonization by the pathogen, as shown by the weak immunohistochemical labelling of *M. bovis* and reduced lung lesions in this group. The lack of positive *M. bovis* immunolabelling despite the presence of an inflammation in bronchioli in the several *M. bovis*-infected calves, might be an effect of the treatments disrupting the bacteria or that *M. bovis* was not present in those lung sections, although it could be present elsewhere. However for the E1 group the results indicate it is due to effective treatment. These results combined with the humoral response in the enrofloxacin-treated animals on provide a clear indication that this treatment; along with the specific calf immune response is a suitable therapeutical approach. However, one aim of this study was to assess and then be able to advise on the most effective and targeted use of antimicrobials (and other drugs) to control *M. bovis* infections. Work is also required to develop alternative therapies to comply with guidelines for the prudent use of antimicrobials in veterinary medicine [16]. This may be based on other antimicrobials that are potentially effective against cattle mycoplasmas possibly combined with adjunctive components (expectorantia, bronchodilators, etc.) or development of a specific *M. bovis* vaccine.

Acute phase proteins (APPs) are known as biomarkers of many disorders in cattle, including *M. bovis* infections [17–20]. The beneficial effect of treatment with enrofloxacin alone was more apparent in selected APP concentrations, mainly in the Hp changes, in restoring the disturbed homeostasis following the *M. bovis* infecting dose. Cytokines such as IL-1β and TNF-α are known as key mediators of inflammation and APP production [17]. It appears that flunixin that was used in the E2 and E3 groups had an inhibitory effect on production of these cytokines. However this was probably more related to the anti-inflammatory properties of this drug rather than real therapeutic effect. Some cytokine suppression was observed in the E1 group, however a general stabilization of the other cytokines examined was shown.

Enrofloxacin given alone caused strong immune response and visibly reduced clinical changes and lung lesions in the *M. bovis* infected calves. Histopathological results supplemented with immunohistochemistry findings also confirmed the effectiveness of this therapy. In contrast flunixin co-administration was ineffective as the *M. bovis* infection manifested with clinical respiratory signs and more severe lung pathology. Addition of pegbovigrastim injection in enrofloxacin-flunixin-treated calves may have exacerbated the disease possibly due to drug interactions.

## Conclusion

The results of immunological, clinical, gross pathology, histopathological and IHC examinations demonstrated that antimicrobial therapy, in this case, enrofloxacin, given alone is more effective for treating *M. bovis* infected calves than combination therapy. Therefore the co-administration of flunixin meglumine or flunixin meglumine and pegbovigrastim with some antimicrobials, specifically enrofloxacin, to treat *M. bovis* infections study should be avoided.

## Methods

Experimental procedures and animal management protocols were carried out in accordance with the detailed unified requirements of the Local Ethics Committee on Animal Experimentation, which also meet the EU standards.

### Animals

Twenty eight, four-week old clinically healthy female calves were purchased from local farms from herds previously known to be free of *M. bovis* and then delivered to the Institute’s vivarium and observed during an adaptive period of 3 weeks. Each group were housed in separate pens, in a shared air space, fed milk replacer, hay and water ad libitum. Prior to the experiment the calves were examined for *M. bovis* antigen and antibody using Monoscreen AgELISA *Mycoplasma bovis* and Monoscreen AbELISA *Mycoplasma bovis* (BIO K 341/2 and BIO K 260/2 respectively) (Bio-X Diagnostics, Belgium). Deep nasal swabs were examined to exclude the presence of *Pasteurellaceae* species by culturing on blood agar, nutrient agar and nutrient agar with glucose at 37 °C. The culture plates were examined daily for 3 days for typical *Pasteurellacae* colony morphology. Any suspect colonies were subjected to bacteriological identification tests as detailed in [21]. Antibodies to the bovine respiratory viruses BHV1, BVDV, BRSV and PI3V were assayed using ELISA (Bio respiratory ELISA kit Pentakit BIO K 028/2, Bio-X Diagnostics, Belgium).

### Antimicrobial testing

*Mycoplasma bovis* strain KP795974 which was originally isolated from the milk of a Polish mastitic cow had previously been used to establish a successful calf challenge model (which produced typical *M. bovis* infected gross lung morphology within 3 weeks) [4]. The minimal inhibitory concentrations (MICs) of this strain was determined against 12 antimicrobials by the microbroth dilution method using “Sensititre” plates [6]. Enrofloxacin was subsequently selected to treat the calves in this experiment.

### Calf challenge

The field *M. bovis* strain KP795974 was used to infect 24 calves. A dose of 23 ml of 1.5 × 10^8^ CFU/ml was given at experiment day 0 when calves were first infected and then at 48 h intervals, twice intratracheally up to the marginal lobe of right lung and once by aerosol application into the animal nostrils. The inoculum was prepared as described and used successfully previously [22]. Four negative control (NC) calves were similarly treated with phosphate buffered saline (PBS) pH 7.2.

### Sample collection

Blood samples (a maximum of 26 ml) and deep nasal swabs were collected from each animal daily up to day 9 post the first infecting dose. Subsequently these samples were collected weekly.

### Calf treatment

At day 9 post the first infection the experimental calves (*n* = 24) were allocated into four randomised subgroups named E1, E2, E3 and positive control (PC). The E1, E2 and E3 calves were treated subcutaneously with enrofloxacin (Enflocyna®, Biowet Pulawy) at the dose of 5 mg/kg body weight (b.w.) once a day for three consecutive days as per the manufacturer’s recommendations.

In addition the E2 and E3 group of calves received flunixin meglumine (Finadyne® Solution, MSD Animal Health) administered intravenously at the dose of 2.2 mg of flunixin/kg b.w. once a day for three consecutive days.

The E3 calves were also treated with pegbovigrastim injection (Imrestor®, Elanco) which was administered subcutaneously at the dose of 2.8 mg/calf once and then repeated after 7 days (day 16 post infection).

The PC group were not treated.

### Clinical observations

Rectal temperature and clinical signs of *M. bovis* respiratory infection such as nasal discharge, cough, respiratory murmurs, dyspnoea etc. were observed in the early morning before calf feeding and recorded in all calves for experimental days 0 to 9 and on days 10, 11, 16, 23 and 30.

### Necropsy

On experiment day 30, 3 weeks post the first calf treatment all calves from E1, E2, E3 and PC groups and two NC calves were euthanased using anesthetic - pentobarbital administered intravenously once at the dose of 140 mg/kg b.w. without previous premedication. Lung, mediastinal lymph node (MLN) and trachea samples were collected for further analyses. The remaining two NC calves were transfered to other experiments according to the Local Ethics Committee on Animal Experimentation permission.

### *Mycoplasma bovis* antigen

#### Antigenic ELISA

The nasal swabs and post mortem collected organs were examined for *M. bovis* antigen detection by ELISA as described previously. Optical densities were measured at OD_450_. The *M. bovis* antigen detection for each sample was expressed as a percentage (Val) and calculated by dividing the signal read for each sample well by the corresponding positive control signal and multiplying by 100. The sample was interpreted as positive if Val was greater than 7% as per manufacturers recommendations.

### Immunohistochemistry

For *M. bovis* antigen detection in the lung tissues, an immunohistochemical analysis was performed. The sections were deparaffinised in xylene, re-hydrated in descending concentrations of ethanol and incubated for 10 min in 3% H_2_O_2_ diluted in methanol to block endogenous peroxidase activity, then submitted to heat-induced antigen retrieval by incubation in citrate buffer at pH 6.0 for 20 min. The slides were then incubated for 1 h with monoclonal mouse antibody against *M. bovis* (Anti-Mycoplasma bovis antibody, cat. no. MAB970, Millipore, USA, dilution 1:2000) as a primary antibody. The detection of the antibody was performed using Dako REAL EnVision Detection System, Peroxidase/DAB, Rabbit/Mouse (K5007, DAKO, Denmark), involving incubation with a peroxidase-conjugated polymer as a secondary antibody (for 30 min) and DAB+ Chromogen applied for visualisation of the reaction. Sections were counterstained with Mayer’s haematoxylin, dehydrated and mounted. Sections incubated with PBS instead of the primary antibody were used to confirm specificity of the staining. The tissues were analysed under a light microscope for the presence of brown staining indicating positive labelling of *M. bovis* antibody.

### Anti-*Mycoplasma bovis* specific antibodies

The blood samples were tested for specific anti-*M. bovis* antibodies using the ELISA kit as described previously. Optical densities were measured at OD_450_. The *M. bovis* antibody level for each sample was expressed as a percentage (Val) and calculated by dividing the signal read for each sample well by the corresponding positive control serum signal and multiplying by 100. The sample was interpreted as positive if Val was greater than 37% as per manufacturers recommendations.

### Immunological analyses

The blood samples were examined using commercial ELISA kits according to the manufacturer’s instructions for the following parameters: a concentration of acute phase proteins (APPs) including serum amyloid A (SAA; Tridelta Development Ltd.), haptoglobin (Hp; Tridelta Development Ltd.), alpha-2-Macroglobulin (A2M; Cusabio) and albumin (Alb, BSA; Cloud-Clone Corp.) as well as cytokine activity such as IL-1, IL-2, IL-10 and TNF-α (Cloud-Clone Corp.).

The optical densities in the microwells for APP and cytokine concentrations as well as for the antibodies to *M. bovis* and *M. bovis* antigen detection were read by an automated plate reader (Elx800 Microplate Reader, BioTek Instruments, Inc., USA) using the KC Junior programme (BioTek Instruments, Inc., USA).

### Gross pathology

At post mortem gross pathology was observed and recorded with particular regard to: colour and structure, presence of fibrin, caseous mass, abscesses and consolidation (lung); size and structure of MLN; and presence, type and amount of inflammatory exudate (trachea).

### Routine optical microscopy examination

All post mortem samples underwent histopathological examination. Tissue samples were fixed in 10% neutral-buffered formalin, then routinely processed into paraffin blocks and sectioned at 5 μm. The slides were stained with hematoxylin and eosin (HE) and analysed for a presence of morphological changes using light microscopy.

### Statistical analysis

The results are presented as arithmetic means ± standard deviation. The differences between the mean values recorded in the E1, E2 and E3 groups and both control groups (PC and NC) at the same time point were analysed using *t* test with a statistically significant level of *P* < 0.05.

## Supplementary information

**Additional file 1: Table S1.** The clinical observations for the experimental (E1, E2, E3) and control calves post the first infecting dose of *M. bovis*.

**Additional file 2: Table S2.** The clinical observations for the experimental (E1, E2, E3) and control calves post day of therapy starting.

## Data Availability

All the data supporting our findings is contained within manuscript. The raw datasets analysed during the current study are available from the corresponding author on reasonable request.

## Abbreviations

A2M: Alpha-2-macroglobulin
APPs: Acute phase proteins
b.w.: Body weight
BALT: Bronchiolar-associated lymphoid tissue
BRD: Bovine respiratory disease
BSA: Albumin
E1, E2, E3: Experimental subgroup 1, 2 and 3, respectively
HE: Hematoxylin and eosin
Hp: Haptoglobin
*M. bovis*: *Mycoplasma bovis*
MICs: Minimal inhibitory concentrations
MLN: Mediastinal lymph node
NC: Negative control
NSAIDs: Nonsteroidal anti-inflammatory drugs
PBS: Phosphate buffered saline
PC: Positive control
SAA: Serum amyloid A
Val: Percentage
