# Supplementary material for: Preliminary study on the effects of enrofloxacin, flunixin meglumine and pegbovigrastim on Mycoplasma bovis pneumonia
Source: BMC Vet Res. 2019 Oct 26;15:371. doi: 10.1186/s12917-019-2122-3 (PMC6815429; doi:10.1186/s12917-019-2122-3)
Supplement: Supplementary file 1 — Additional file 1: Table S1. The clinical observations for the experimental (E1, E2, E3) and control calves post the first infecting dose of M. bovis. [file 12917_2019_2122_MOESM1_ESM.docx]

Table S1. The clinical observations for the experimental (E1, E2, E3) and control calves post the first infecting dose of *M. bovis*.

|  | Rectal Temperature | | | | | Nasal discharge | | | | | Cough | | | | | Crackles | | | | | Dyspnoea | | | | | Others | | | | |
| --- | --- | --- | --- | --- | --- | --- | --- | --- | --- | --- | --- | --- | --- | --- | --- | --- | --- | --- | --- | --- | --- | --- | --- | --- | --- | --- | --- | --- | --- | --- |
| Post the first infecting dose of  *M. bovis* | NC | PC | E1 | E2 | E3 | NC | PC | E1 | E2 | E3 | NC | PC | E1 | E2 | E3 | NC | PC | E1 | E2 | E3 | NC | PC | E1 | E2 | E3 | NC | PC | E1 | E2 | E3 |
| Day 1 | N | N | 1 incr | 1 incr | 1 incr | N | N | 5 | 4 | 2 | N | 2 | N | 1 | 1 | N | N | N | N | N | N | N | N | N | N | N | N | N | 1 CD | N |
| Day 2 | N | 1 incr | 1 incr | N | 1 incr | N | 4 | 2 | 5 | 5 | N | N | N | N | N | N | N | N | N | N | N | N | N | N | N | N | 1 CD | N | 1 CD | N |
| Day 3 | 1 incr | 1 incr | 1 incr | 1 incr | N | N | 3 | 4 | 4 | 4 | N | N | N | N | N | N | N | N | N | N | N | N | N | N | N | N | 1 CD | N | N | N |
| Day 4 | N | N | 2 incr | 2 incr | N | N | 4 | 2 | 5 | 4 | N | N | N | N | N | N | N | N | N | N | N | N | N | N | N | N | 1 CD | N | N | N |
| Day 5 | N | 2 incr | 1 incr* | 1 incr* | 1 incr | N | 2 | 6 | 5 | 4 | N | 1 | N | N | N | N | N | N | N | N | N | N | N | N | N | N | 1 H | N | N | N |
| Day 6 | N | 2 incr | 1 incr | N | 2 incr | N | 4 | 5 | 4 | 5 | N | N | N | N | N | N | N | N | 1 | N | N | N | N | N | N | N | 1 CD | N | N | N |
| Day 7 | N | 2 incr  (1 incr*) | 1 incr | 1 incr* | 2 incr | N | 5 | 4 | 6 | 5 | N | 1 | 3 | N | N | N | 1 | N | N | N | N | 1 | N | N | N | N | 1 CD  PP/DM/H  (the same calf) | N | N | N |
| Day 8 | N | 2 incr | 2 incr  (1 incr*) | 1 incr* | 1 incr | N | 5 | 6 | 4 | 5 | N | 2 | 2 | 1 | 1 | N | 1 | N | N | N | N | 1 | 1 | N | N | N | 1 CD  PP/DM/H (the same calf) | 1 S | N | N |
| Day 9 | N | 2 incr | 3 incr  (1 incr*) | 1 incr* | 2W | N | 5 | 6 | 5 | 6 | N | 1 | 1 | N | N | N | 1 | N | N | N | N | 1 | 1 | N | N | N | 1 CD  PP/DM/H (the same calf) | 1 S | N | N |
| Total | 1 | 12 | 13 | 8 | 10 | 0 | 32 | 40 | 42 | 40 | 0 | 7 | 6 | 2 | 2 | 0 | 3 | 0 | 0 | 0 | 0 | 3 | 2 | 0 | 0 | 0 | 11 | 2 | 2 | 0 |

NC, negative control; PC, positive control; E1, group received antibiotic alone; E2, group received antibiotic combined with NSAID; E3, group received antibiotic combined with NSAID and pegbovigrastim injection; N, normal.; Incr, increase > 39.5 °C; *, increase ≥ 41 °C; CD, conjunctiva discharge; PP, preferential posture; DM, dry muzzle;
H, hypophagia/hypodipsia; S, stasis.

Total of six calves per PC, E1, E2 and E3 groups.

Total of four calves per NC group.
