# Supplementary material for: Preliminary study on the effects of enrofloxacin, flunixin meglumine and pegbovigrastim on Mycoplasma bovis pneumonia
Source: BMC Vet Res. 2019 Oct 26;15:371. doi: 10.1186/s12917-019-2122-3 (PMC6815429; doi:10.1186/s12917-019-2122-3)
Supplement: Supplementary file 2 — Additional file 2: Table S2. The clinical observations for the experimental (E1, E2, E3) and control calves post day of therapy starting. [file 12917_2019_2122_MOESM2_ESM.docx]

Table S2. The clinical observations for the experimental (E1, E2, E3) and control calves post day of therapy starting.

|  | Rectal Temperature | | | | | Nasal discharge | | | | | Cough | | | | | Crackles | | | | | Dyspnoea | | | | | Others | | | | |
| --- | --- | --- | --- | --- | --- | --- | --- | --- | --- | --- | --- | --- | --- | --- | --- | --- | --- | --- | --- | --- | --- | --- | --- | --- | --- | --- | --- | --- | --- | --- |
| Post day of therapy starting | NC | PC | E1 | E2 | E3 | NC | PC | E1 | E2 | E3 | NC | PC | E1 | E2 | E3 | NC | PC | E1 | E2 | E3 | NC | PC | E1 | E2 | E3 | NC | PC | E1 | E2 | E3 |
| Day 1 (10) | N | 1 incr | 1 incr | N | N | N | 6 | 4 | 3 | 3 | N | 1 | N | N | N | N | 1 | N | N | N | N | 1 | 1 | N | N | N | 1 CD  PP/DM/H (the same animal)  2 S | 1 S | N | N |
| Day 2 (11) | N | 1 incr | N | 1 incr* | N | N | 5 | 3 | 4 | 2 | N | N | N | N | N | N | N | N | N | N | N | N | 1 | N | N | N | 1 death  1 CD  1 S | 1 S | N | N |
| Day 7 (16) | N | N | 2 incr | 1 incr | 2 incr | N | 4 | 3 | 4 | 4 | N | 1 | N | N | N | N | N | N | N | N | N | N | N | N | N | N | 1 CD  1 S | N | N | 1 PP |
| Day 14 (23) | N | N | N | 1 incr | 3 incr | N | 3 | 1 | 3 | 2 | N | N | N | N | N | N | N | N | N | N | N | N | 1 | N | N | N | 1 CD 1 S | N | N | 1 PP |
| Day 21 (30) | N | N | 1 incr | N | N | N | 3 | 1 | 2 | 1 | N | N | N | N | N | N | N | N | N | N | N | 1 | N | N | N | N | 1 CD  1 PP | N | N | 1 PP |
| Total | 0 | 2 | 4 | 3 | 5 | 0 | 21 | 12 | 16 | 12 | 0 | 2 | 0 | 0 | 0 | 0 | 1 | 0 | 0 | 0 | 0 | 2 | 3 | 0 | 0 | 0 | 13 | 2 | 0 | 3 |

NC, negative control; PC, positive control; E1, group received antibiotic alone; E2, group received antibiotic combined with NSAID; E3, group received antibiotic combined with NSAID and pegbovigrastim injection; N, normal.; Incr, increase > 39.5 °C; *, increase ≥ 41 °C; CD, conjunctiva discharge; PP, preferential posture; DM, dry muzzle;
H, hypophagia/hypodipsia; S, stasis. () – experiment day.

Total of six calves per PC, E1, E2 and E3 groups.

Total of four calves per NC group.
